# Structure and Oligonucleotide Binding Efficiency of Differently Prepared Click Chemistry-Type DNA Microarray Slides Based on 3-Azidopropyltrimethoxysilane

**DOI:** 10.3390/ma14112855

**Published:** 2021-05-26

**Authors:** Emilia Frydrych-Tomczak, Tomasz Ratajczak, Łukasz Kościński, Agnieszka Ranecka, Natalia Michalak, Tadeusz Luciński, Hieronim Maciejewski, Stefan Jurga, Mikołaj Lewandowski, Marcin K. Chmielewski

**Affiliations:** 1 Poznań Science and Technology Park, Adam Mickiewicz University Foundation, Rubież 46, 61-612 Poznań, Poland; emilia.frydrych@ppnt.poznan.pl (E.F.-T.); maciejm@amu.edu.pl (H.M.); 2 Institute of Bioorganic Chemistry, Polish Academy of Sciences, Noskowskiego 12/14, 61-704 Poznań, Poland; tomasz.r.ratajczak@gmail.com; 3 Institute of Molecular Physics, Polish Academy of Sciences, M. Smoluchowskiego 17, 60-179 Poznań, Poland; kos55@wp.pl (Ł.K.); amarczynska1@wp.pl (A.R.); michalak@ifmpan.poznan.pl (N.M.); lucinski@ifmpan.poznan.pl (T.L.); 4 Faculty of Chemistry, Adam Mickiewicz University, Uniwersytetu Poznańskiego 8, 61-614 Poznań, Poland; 5 NanoBioMedical Centre, Adam Mickiewicz University, Wszechnicy Piastowskiej 3, 61-614 Poznań, Poland; stjurga@amu.edu.pl

**Keywords:** DNA microarrays, click chemistry, organofunctional silanes, surface modification, functional layers, structure, atomic force microscopy (AFM), X-ray photoelectron spectroscopy (XPS)

## Abstract

The structural characterization of glass slides surface-modified with 3-azidopropyltrimethoxysilane and used for anchoring nucleic acids, resulting in the so-called DNA microarrays, is presented. Depending on the silanization conditions, the slides were found to show different oligonucleotide binding efficiency, thus, an attempt was made to correlate this efficiency with the structural characteristics of the silane layers. Atomic force microscopy (AFM), X-ray photoelectron spectroscopy (XPS) and X-ray reflectometry (XRR) measurements provided information on the surface topography, chemical composition and thickness of the silane films, respectively. The surface for which the best oligonucleotides binding efficiency is observed, has been found to consist of a densely-packed silane layer, decorated with a high-number of additional clusters that are believed to host exposed azide groups.

## 1. Introduction

DNA microarrays are ordered matrices of oligonucleotides or larger deoxyribonucleic acid (DNA) fragments attached to activated solid surfaces, usually in the form of glass slides [1]. They enable high-throughput screening of biomolecular interactions, allowing the analysis of the level of gene expression, studies of the genome structure, identification of a genetic polymorphism or detection of viral, bacterial and fungal pathogens. What is more, microarray experiments require minimum amounts of samples, which is an important factor in genetic studies. Thanks to this, microarrays became essential tools in many research areas, such as medicine, pharmacology, biochemistry, genetics and microbiology. As far as the gene expression is concerned, a single microarray chip may allow monitoring large numbers of single nucleotide polymorphisms in the genome of an organism, using base-pairing (A–T and G–C for DNA; A–U and G–C for RNA) as the underlying principle.

Recently, many variations of microarray formats have been developed. An interesting example which allows studying the gene functions are cell-based microarrays, where cells are immobilized either on a flat substrate or on particles. Such arrays allow exploring the role of genes in specific cell biological processes and disease states (the cells can express encoded proteins at the site where the genes are deposited, while overexpression can induce a specific modification in the biology of the transfected cells [2]). Microarray technology can be also utilized in the studies on gene therapy, which is one of our scientific interests [3]. In this respect, the RNA interference (RNAi) technology, which we also use [4], can be a powerful means for investigating the gene functions using cell-based microarrays.

In a classical array, the probes are arranged sequentially to provide a platform for detecting known/unknown DNA samples [5]. Owing to this, the technology enables identifying important genes involved in the physiopathology of diverse diseases, including cancer. Microarrays utilizing oligonucleotide or DNA probes are usually prepared using one of the two approaches: (i) in situ oligonucleotide synthesis [6,7], which is a chemical, multistep synthesis directly at the solid surface, or (ii) mechanical deposition of DNA (synthesized by the phosphoramidite approach on a CPG-solid support) by means of spotting, ink-jet printing or the split-pin technology [8,9,10,11]. Traditional spotting techniques have limitations coming from the drop and needle sizes which, in turn, limit the size of microarrays. Thus, miniaturization of microarrays requires smaller and smaller spotting elements to anchor the nucleic acid in a form that is as compact as possible and a permanent link between the probe and the surface [12].

Immobilization of probes at the surface of a microarray slide can be based on electrostatic interactions or a covalent bonding [13]. Although the latter requires the functionalization of DNA with an active linker, it seems to be the simplest and most precise immobilization way [14]. The type of the linker used is determined by the functional groups at the solid support [15]. Therefore, it is desirable to design chemical linkers, allowing permanent attachment of nucleic acids at solid surfaces. In 2000, Sharpless et al. have developed “click” chemistry as a modular approach to irreversibly couple two molecules under mild reaction conditions [16,17]. Thanks to its biocompatibility, click chemistry has become an interesting approach to DNA immobilization on solid supports using micro-contact printing [18]. Among the various types of click reactions, the most commonly used for the DNA immobilization is the 1,3-dipolar azide-alkyne cycloaddition catalyzed by copper ions (CuAAC) [19,20]. Azides and alkynes are relatively easy to introduce to biomolecules and they are inert under physiological conditions. Moreover, triazoles (linkers produced by the click approach) are very stable, being almost impossible to oxidize or reduce [21,22,23].

Several years ago, we showed that CuAAC reaction can be applied as a method for the preparation of DNA microarrays through oligonucleotides immobilization on glass supports surface-functionalized using 3-azidopropyltrimethoxysilane (AzPTMS) [24]. For this purpose, the nucleic acids probes are modified with a pentynyl linker. During these studies, we observed that different silane concentrations and application methods (incubation or sonication) led to diverse oligonucleotide binding efficiency. Here, we present the results of a structural study on the AzPTMS-modified glass plates aimed at correlating the structural characteristics of differently-prepared silane layers with their efficiency in anchoring oligonucleotide probes. The surface topography, chemical composition and thickness of silane films are determined using atomic force microscopy (AFM), X-ray photoelectron spectroscopy (XPS) and X-ray reflectometry (XRR), respectively. The binding efficiency is presented through the analysis of fluorescent images of as-printed, as well as additionally washed, DNA microarrays.

## 2. Materials and Methods

### 2.1. Materials

The key materials used for the studies were acquired from the following sources: 4-pentyl-1-ol, sodium ascorbate, triethylamine and sodium dodecyl sulfate (SDS) were purchased from Sigma-Aldrich (Darmstadt, Germany). 4-dimethylaminopyridine (98%) was acquired from Fluka (Buchs, Switzerland). Buffer SpotQC was obtained from Integrated DNA Technologies (Coralville, IA, USA) and saline-sodium citrate (SSC) was from Ambion (Austin, TX, USA). A total of 4% Trilux, from Analab (Warsaw, Poland), was used as a detergent. All organic solvents for glass slides preparation were purchased from Avantor Performance Materials Poland S.A. (formerly POCH, Gliwice, Poland) and used as purchased. Nitrogen gas (99.999%) was acquired from Linde (Munich, Germany). Redistilled water was obtained from a glass water distiller system type 2304 from GFL Gesellschaft für Labortechnik GmbH (Berlin, Germany). Glass slides were the standard 26 × 76 × 1 mm soda lime microscope slides supplied by Knittel Glass (Braunschweig, Germany). Silica gel chromatography was conducted with the use of the 70–230 mesh silica gel from Merck (Darmstadt, Germany). Phosphoramidite reagents for the DNA synthesis were purchased from Glen Research (Sterling, VA, USA), fluorophore CPG with Quasar 670 from Biosearch Technologies (Novato, CA, USA) and solvents from Carl Roth GmbH (Karlsruhe, Germany).

### 2.2. Synthesis of 3-Azidopropyltrimethoxysilane (AzPTMS) and Functionalization of Glass Slides

AzPTMS was synthesized following the procedure developed by Paoprasert et al. [25]. Briefly, a mixture of 3-chloropropyltrimethoxysilane (15 mL, 82 mmol) and sodium azide (10.5 g, 162 mmol) in N,N-dimethylformamide (100 mL) was heated under reflux and, subsequently, stirred for 23 h at 95 °C. Then, it was distilled under reduced pressure to obtain 3-azidopropyltrimethoxysilane in the form of a colorless liquid (14.03 g, 86% yield). A successful synthesis was confirmed with the use of Fourier transform infrared spectroscopy (FT-IR; Bruker Tensor 27 spectrometer equipped with a SPECAC Golden Gate diamond ATR unit), as well as nuclear magnetic resonance (NMR; Bruker Advance DRX 400 spectrometer operating at frequencies of 400.13201 MHz (^1^H), 100.62281 MHz (^13^C) and 79.45750 MHz (^29^Si)). The results are presented in Figures S1–S4. Prior to silanization, the glass slides were flushed thoroughly with acetone, dried in a stream of nitrogen, washed with a detergent, rinsed several times with distilled and filtered water for 10 min and dried again in nitrogen. Next, they were immersed in toluene solutions of AzPTMS with a given concentration, either 0.5% or 2%, and kept for 1 h (which is further denoted as the “incubation” procedure) or sonicated in an ultrasonic bath (Elma Elmasonic S 60H) for 30 min (“sonication”). As shown in the work published by some of us [24], during the silanization, the slides become covered with a silane layer exhibiting azide functional groups, thus becoming convenient substrates for anchoring molecules through the click reaction. The slides were then sonicated in a fresh portion of toluene for 10 min, placed in an oven set at 120 °C and baked for 1 h. After cooling down to room temperature, they were rinsed with toluene and dried in a stream of nitrogen. Following the preparation, the slides were stored in a nitrogen dry box to prevent deposition of contaminants at the surface.

The preparation of a thin and uniform silane layer at a glass support is a challenging task. The silanization process can be influenced by many factors, such as the silane concentration, solvent used, water content, reaction time and temperature. Improper selection of these parameters may result in the formation of a discontinuous film. Thicker films, on the other hand, tend to exhibit three-dimensional agglomerate structures or multilayer islands, which may lead to a variable performance in the oligonucleotides binding, as well as the appearance of a strong background signal in microarray experiments, through undesired fluorescence. During our preliminary studies, partially described in References [24,26], we tried modifying glass slides using different silane concentrations in toluene: 0.02%, 0.2%, 0.5%, 5% and 10%. For the concentration of 0.02%, no immobilization of oligonucleotide probes was observed. The concentrations of 5% and 10%, on the other hand, resulted in silane condensation at the surface and pronounced fluorescent background in microarray experiments. As far as the silanization method is concerned, both incubation and sonication were tested. Initially, the slides were incubated for 2 h, however, the time was shortened to 1 h due to the lack of the observed difference in effectiveness of oligonucleotides binding. In the case of sonication, prolonged exposure to ultrasonic waves caused the water in the washer, and thus the silane solution, to heat up. On this basis, the optimum sonication time of 30 min was established. Therefore, within the present study, we compared two concentrations: the 0.5% and a slightly higher one, i.e. 2%, that was still expected to provide a low background level. We also used both silanization methods: incubation and sonication.

### 2.3. Contact Angle Measurements

Static contact angle measurements were carried out using a Krüss DSA 100E auto goniometer. The studies were performed for clean and silanized glass slides and aimed at determining the surface wettability. Analyses were made on sessile drops of water (5 µL droplets, with a dosing rate of 100 µL/min) by measuring the tangent to the drop at its intersection with the slide’s surface in an image analysis software. The contact angles values were determined by performing a minimum of eight independent measurements for each slide and are presented as a mean value ± standard deviation.

### 2.4. Atomic Force Microscopy (AFM)

AFM studies were performed in air for a clean glass slide, slides covered with silane layers prepared using 0.5% and 2% AzPTMS concentrations and a slide with imprinted nucleic acid probes. The measurements were carried out to determine the surface topography and roughness of the slides, the thickness of the silane layer (where possible) and the morphology of the attached nucleic acid probes. The microscope, Bruker Dimension Icon AFM, was working in the tapping mode (the so called PeakForce Tapping “ScanAsyst” mode). The images were acquired using commercial probes (Bruker “ScanAsyst-Air”) consisting of a symmetrical Si tip with a 2 nm radius attached to a 600 nm-long silicon nitride cantilever with a resonant frequency of ~70 kHz and a spring constant of 0.4 N/m. The results were processed using the Gwyddion 2.51 64-bit computer software.

### 2.5. X-ray Photoelectron Spectroscopy (XPS)

XPS measurements were aimed at determining the chemical composition of the silane layer. The studies were performed under ultra-high vacuum (UHV; base pressure: 6 × 10^−10^ mbar) using a non-monochromatic Al K_α_ (1486.6 eV) X-ray radiation source (PREVAC RS 40B1) and a hemispherical electron energy analyzer (VG Scienta R3000). The spectra were recorded at room temperature using a pass energy (PE) of 200 eV. Each spectrum represents the average of 20 sweeps taken with a 0.5 eV step. The obtained data were normalized to the highest peak (O 1s), smoothed (FFT, 5 points) to increase the signal-to-noise ratio and calibrated with respect to the position of the main C 1s component. In the case of a clean glass slide, the main contribution was expected to originate from the sp^2^ (C=C) carbon, therefore, the calibration was made by shifting the main component to 284 eV. In the case of silanized samples, the main contribution was expected from C–H and C–C-bonded carbon in silane molecules, so the main component was shifted to 285 eV. The fittings were made with the CasaXPS Version 2.3.22PR1.0 software (Casa Software Ltd.), using the Voigt function (linear combination of Gauss and Lorentz functions) and a Shirley background subtraction.

### 2.6. X-ray Reflectometry (XRR)

XRR was used to determine the total thickness of the silane layer. The reflectometer (PANalytical) was equipped with a Cu K_α_ radiation source (λ = 1.54 Å) operating at 45 kV and 40 mA. The measurements were performed in air. The results were fitted using the X’Pert Reflectivity software package.

### 2.7. Synthesis of Fluorescently-Labeled Oligodeoxynucleotides (ODNs)

**Figure 1:**
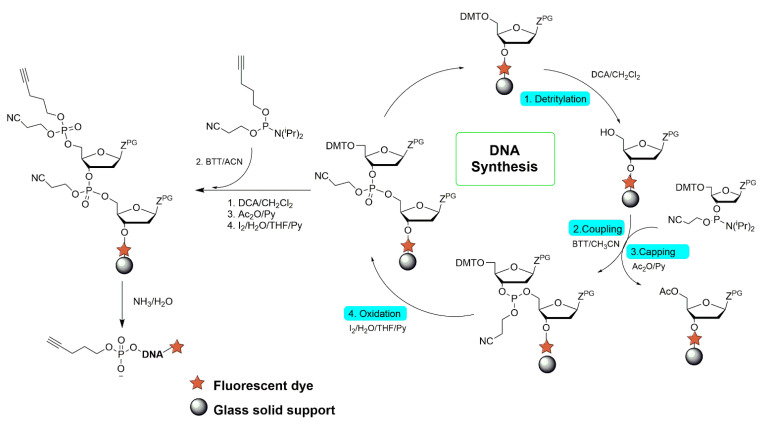
A schematic drawing showing the steps of the synthesis of the DNA containing fluorescently labeled tag at the 3′ end and pentynyl linker at the 5′ end.

The chemical synthesis of DNA was conducted using the K&A Laborgeräte GbR H-4 DNA/RNA synthesizer. The process is schematically shown in Figure 1. In short, a solid-phase syntheses of ODNs with a pentynyl linker were performed from the 3′ to the 5′ direction on a 0.2 μmol scale using commercial nucleoside phosphoramidites. In the last step of the synthesis, a 4-pentynyl-(2-cyanoethyl)-N,N-diisopropylphosphoramidite linker was attached to each ODN at the 5′ end. The compound was obtained according to the procedure described in Ref. [26]. For microarray tests, three types of ODNs were synthesized with the following sequences (where F refers to the pentynyl linker and Q points out the position of the fluorescent tag):

1. ODN-8–5′ F AAC GGA GA Q 3′
2. ODN-12–5′ F AAC GGA GAT GGT Q 3′
3. ODN-16–5′ F AAC GGA GAT GGT TAT TQ 3′

The condensation step was carried out with a coupling time of 60 s. The ODNs were cleaved and deprotected using a mixture of ethanol and ammonium hydroxide (1:3 v:v) for 5 h at room temperature. Purification was performed using denaturing gel electrophoresis. The ODNs extracted from the gel were desalted by passing them through filtration columns (NAP-25, GE Healthcare). The products were lyophilized, resulting in 1.0 OD (ODN-8), 2.2 OD (ODN-12) and 2.6 OD (ODN-16). The purity of ODNs was verified with reverse phase high performance liquid chromatography (HPLC; Shimadzu UFLC Prominence system with a LC-20AD pump and a Clarity 5 μm C(18)2 100 Å column (15 cm × 4.6 mm)). A gradient of acetonitrile 0–60% (B) in ammonium acetate solution (0.05 mol, pH 7) (A) was used for the studies. The DAD detector was set to 254 nm. The results are presented in Figures S5–S7. Starting from 0.01 mol triethylammonium acetate (pH 7.0), a linear gradient 0 to 10% of MeCN was pumped at a flow rate of 0.9 mL/min for 40 min. The obtained oligonucleotides were further analyzed using matrix-assisted laser desorption/ionization mass spectrometry (MS; Bruker microTOF-q system equipped with a time of flight (TOF) detector). Each sample was prepared as follows: 0.1 OD of ODN was mixed directly on an analytical plate with a saturated solution of 2′,4′,6′-trihydroxyacetophenone (1:1 v:v methanol-water), dried in air and analyzed in the negative mode afterwards. The sequences, as well as the calculated and measured atomic mass values, are given in Table 1, while the raw MS spectra are shown in Figures S8–S10.

### 2.8. Immobilization of Modified ODNs on AzPTMS-Functionalized Glass Slides

**Figure 2:**
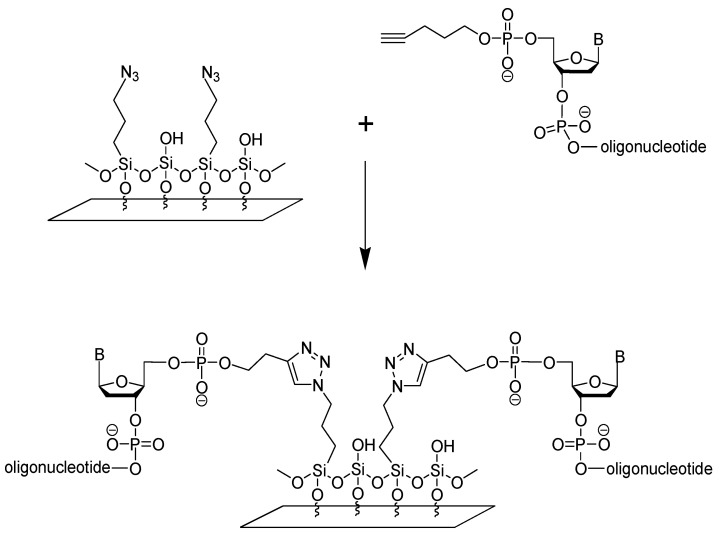
Immobilization of ODNs at the surface of an AzPTMS-functionalized glass slide via “click” chemistry.

The method of ODNs immobilization at the surface of azide-functionalized supports through an alkynyl moiety at the 5′-end of the ODN is a simple, cheap and reliable. Silanes of this type are of bifunctional nature: through the alkoxy group, they can bind to the glass, while the azide group may participate in the click reaction with alkynyl-modified biomolecule (Figure 2). The reactivity of azido functional groups towards alkynyl-terminated oligonucleotides and the stability of the bonding upon washing the slides with imprinted probes could be evaluated thanks to the fluorescent tag at the ODNs’ 3′ ends.

**Figure 3:**
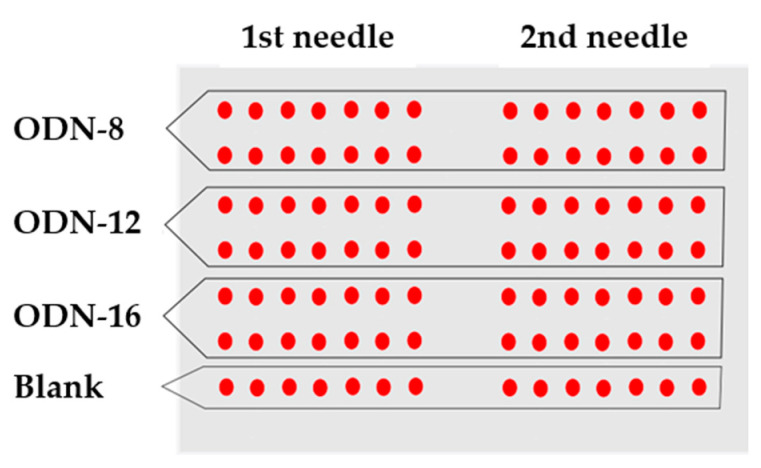
A scheme showing 2-needle printing of DNA microarrays used for the studies. Each probe is spotted at the same concentration in 14 replications by each of the printing needles.

The printing of ODN probes was performed using the Arrayit NanoPrint LM60 spotter and analyzed with the PerkinElmer ScanArray Express microarray scanner, operating at the wavelength of 633 nm. The solutions with oligonucleotides (ODN-8, ODN-12 and ODN-16) were obtained *in situ* by dissolving each probe separately in a proper amount of MilliQ water to obtain 0.1 OD/μL. Each solution was additionally spiked with a solution of sodium ascorbate (24 μL, 0.1 mol), glycerol (10 μL) and copper (II) sulfate (50 μL, 0.04 mol), which resulted in final solutions A_1_, A_2_ and A_3_ used for the printing. To determine the background fluorescence, a negative control, which lacked the fluorescent label, was used. The printing was carried out with two needles following the scheme presented in Figure 3. After spotting, the plates were kept in the microarray printing chamber for 1 h in 70–80% relative humidity at room temperature. Then, they were scanned using a microarray scanner before and after washing with 1% SDS (500 mL) for 1 min at room temperature, as well as MilliQ water (500 mL) for 30 s at 90–100 °C. Finally, the plates were dried by centrifugation.

### 2.9. Molecular Docking Studies

Molecular docking was performed to determine the optimum mutual orientation of silane molecules in two adjacent layers when stacked on top of each other. The computations were carried out using the DOCK 6 computer software. A 3D model of a silane monolayer was constructed using 9 silane molecules. Then, a single silane molecule was docked to the monolayer from the side which hosted azide groups, through the determination of the energetically most preferred adsorption geometry.

## 3. Results and Discussion

Goniometric (contact angle) analysis provides a relatively quick and simple means of assessing the cleanliness of glass slides and the change of their wettability following silanization. The use of water for the measurements made direct correlation between wettability and hydrophilicity/hydrophobicity of the surfaces possible. The contact angles obtained for unmodified glass slides were <10°, indicating a highly-hydrophilic surface. This confirmed that the slides were efficiently cleaned prior to silanization. Modification of the surfaces of the slides using different concentrations of AzPTMS resulted in more hydrophobic surfaces, as manifested by higher contact angles. Table 2 summarizes the contact angle values obtained for clean and differently-prepared slides. According to Collman et al. [27], silanization increases the contact angle and may reach, for the surfaces fully covered with azide groups, 77°. This is in agreement with the values obtained in our experiments. Thus, the results confirmed a successful formation of silane layers.

**Figure 4:**
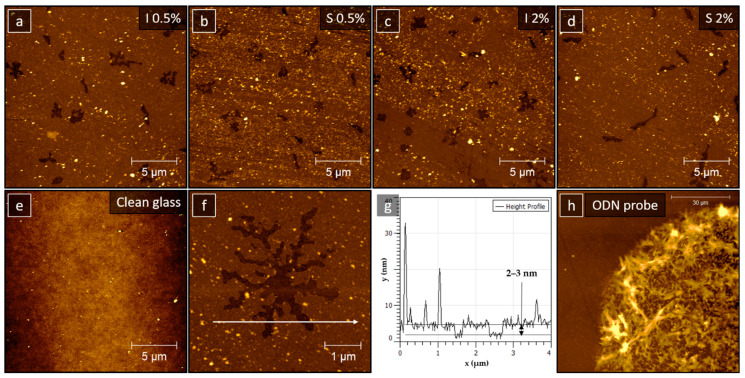
AFM images of silane layers prepared by incubation (**a**,**c**) and sonication (**b**,**d**) in 0.5% (**a**,**b**) and 2% (**c**,**d**) solutions of AzPTMS; (**e**) shows the surface of a clean glass slide; (**f**) shows a zoom-in image to an exemplary hole in the silane layer, while (**g**) the height profile taken over the hole; (**h**) presents an image of an imprinted ODN probe.

AFM measurements were performed to determine the surface topography of silane layers prepared using different silanization processes, i.e. incubation or sonication, as well as different AzPTMS concentrations. Figure 4a,c show AFM images of slides incubated in 0.5% (a) and 2% (c) solutions of AzPTMS, while (b,d) show images of slides sonicated in silane solutions with the same concentrations, respectively. The surface topography of a clean glass slide is shown for comparison in Figure 4e, exhibiting flat surface with no visible structural features. The surface structure of silanized slides differs significantly from clean glass. They are characterized by the presence of clusters and holes, independently of the silanization method and AzPTMS concentration. All the holes observed were of similar depth, ranging from 2 to 3 nm (see the exemplary height profile marked in Figure 4f and shown in Figure 4g). Thus, the results indicated that, following the AzPTMS treatment, glass slides became covered with a thin continuous silane layer, decorated with additional clusters and occasional holes. The clusters were tentatively assigned to silane agglomerates and other adsorbates bonded to the reactive surface either from the solution or from air. Notably, slides prepared by incubation were generally much cleaner than those prepared by sonication (however, it has to be noted that all samples exhibited both clean regions and regions full of adsorbates). Interestingly, the AFM tapping mode phase contrast inside the holes was identical to the contrast on the top of the film (not shown), which indicated that the holes were also filled with silane, i.e. the film had a multilayer structure.

To compare the slides in a quantitative (and not only qualitative) way, we determined the roughness parameters. In the case of inhomogeneous surfaces, like the ones studied in this work, roughness strongly depends on the topography of the microscopic region used for the analysis. In order to obtain reliable results and proper statistics, we used three approaches: (i) from large scale 10 × 10 um^2^ images we cut 2 × 2 um^2^ frames, avoiding very high clusters and holes, measured the roughness of each frame and averaged the results obtained for a given sample; (ii) from each of the images, we cut a single frame—as big as possible to avoid high clusters and holes—and measured its roughness; (iii) we determined the roughness using the whole large scale 10 × 10 um^2^ and 20 × 20 um^2^ images—four for each sample, removed the highest/lowest values and averaged the remaining ones. Using the approaches (i) and (ii), due to small local roughness deviations, it was more reasonable to use the root mean square roughness (R_RMS_) (R_RMS_ value can be easily affected by a single peak or valley). The highest R_RMS_ value was obtained for the slide sonicated in 0.5% solution of AzPTMS, while the lowest one for the slide incubated in 2% AzPTMS. The two remaining samples—the slide incubated in 0.5% AzPTMS and the one sonicated in 2% AzPTMS, were characterized by intermediate roughness values. As far as the average roughness (R_a_) of the slides is concerned, the incubated 2% AzPTMS sample was again characterized by the lowest value, while the other three sample had similar R_a_ values. For the approach (iii), the R_a_ parameter was considered a more reliable one, being not as sensitive to occasional hills and holes. The highest value was again observed for the slide sonicated in 0.5% AzPTMS, while the lowest one for the one incubated in 0.5% AzPTMS. The other two slides were characterized by intermediate R_a_ values. Looking at the R_RMS_ of whole images, the sonicated 0.5% AzPTMS sample was the second-roughest (after the one incubated in 2% AzPTMS, with the remaining two exhibiting much lower roughness values). On the basis on these analyses, it may be concluded that the slide sonicated in 0.5% AzPTMS was generally the roughest one among all the samples studied.

**Figure 5:**
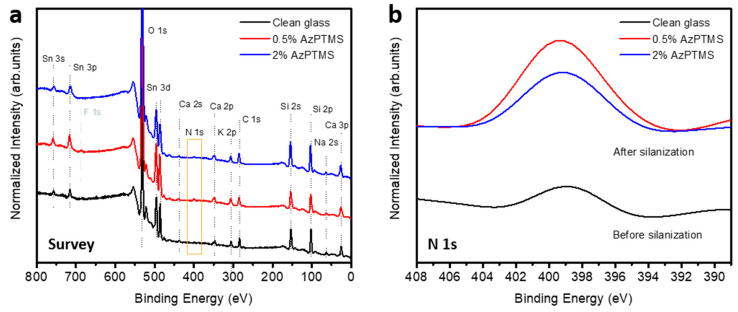
XPS survey (**a**) and N 1s (**b**) spectra obtained for a clean glass slide (black) and slides silanized in 0.5% (red) and 2% (blue) solutions of AzPTMS.

Then, XPS measurements were carried out to compare the chemical structure of the silane layers prepared using different AzPTMS concentrations. For this purpose, it was essential to first establish the elemental composition of a clean glass slide. Figure 5 presents survey (a) and N 1s (b) XPS spectra obtained for an exemplary glass slide (black lines) and slides silanized in 0.5% (red) and 2% (blue) solutions of AzPTMS. The clean slide was composed of Si, O, Na, K, Ca and Sn (most probably originating from the glass production process). The spectra also showed traces of C and N (~399 eV), which are the typical contaminants observed for samples exposed to air. The determined composition is in agreement with the one provided by the manufacturer of the slides in the specification sheet. Following silanization, a significant increase in the nitrogen content was observed (orange frame in Figure 5a), indicating the formation of a silane layer. The concentrations of other elements were slightly varying, which is related to the amorphous and inhomogeneous structure of glass. The highest N 1s signal was observed for the slide silanized in 0.5% AzPTMS (Figure 5b). The recorded peak was also broader than the one obtained for the 2% slide, with the maximum shifted towards higher binding energy values. Based on the model shown in Figure 2, the main expected contribution to the signal was from azide groups (R−N=N^+^=N^−^) which gives rise to three N 1s components corresponding to differently-coordinated nitrogen atoms. The most characteristic component, originating from the middle electron-deficient N atom, appears at the binding energy value of 403–404.4 eV [18,27,28,29,30,31]. The separation between this component and the lowest binding energy one is typically 4–5 eV. Using the well-established N 1s full-with-at-half maximum values of 1.8–2.1 eV and identical peak areas, the 0.5% spectrum could be fitted with at least five components, the outermost ones being separated by approximately 5 eV. The 2% spectrum, on the other hand, could be fitted using four components, with the separation between the lowest and highest ones amounting to 3.5 eV. Precise fitting of the spectra and determination of the nature of all nitrogen-containing groups contributing to them is not trivial (therefore, we do not present the fittings here). However, from the separation of roughly-fitted highest and lowest binding energy components, only the 0.5% slide seems to host a detectable amount of azide groups. Looking at the literature reports [18,27,28,29,30,31], it is evident that the middle N atom in the azide groups may appear in XPS as a distinct (i.e. separated from other N 1s components) well-resolved peak, centered at around 404 eV [27,29,30], but may also give rise to a less-pronounced component positioned at ~403 eV and partially overlapping with other N 1s signals [18,28,31]. This is, most probably, related to the different charge redistribution within the azide groups, which depends on their chemical surrounding. Independently of that, both types of groups were shown to be active in the click reactions (during the reaction, azides transform into triazoles, which are characterized by two peaks located in the 400–402 eV binding energy range). When it comes to our results, the data obtained for the silane layer prepared using 0.5% AzPTMS concentration are very similar to that obtained by Rozkiewicz et al. [18] for an almost identical material system and hosting click-active azide groups.

**Figure 6:**
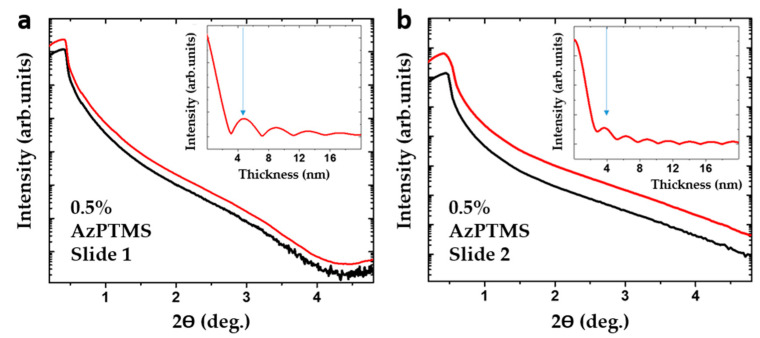
Fitted XRR curves obtained for two different glass slides silanized in a 0.5% solution of AzPTMS. The measured data are presented in black, while the simple fit data are shown in red. The insets show the FFT analysis of the data, where the arrow indicates the determined thickness of the silane layer: 4.6 ± 1 nm (**a**) and 3.9 ± 0.6 nm (**b**).

In order to determine the total thickness of the 0.5% silane layer, XRR measurements were performed. Two different slides prepared in a similar way were analyzed. Fittings of the XRR data, shown in Figure 6a,b, reveal that the total thickness of the respective layers is 4.6 ± 1 (a) and 3.9 ± 0.6 nm (b). This roughly corresponds to twice the thickness observed with AFM, confirming that the silane film consists of two densely-packed layers stacked on top of each other. The thickness of a single layer, approx. 2–3 nm, indicates that in the growth direction, such a layer is composed of more than one silane molecule.

**Figure 7:**
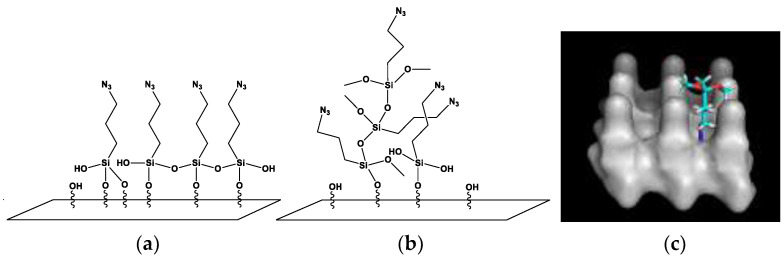
Possible structural arrangements within a polysiloxane layer formed through the reaction of organosilane with a hydroxylated surface: (**a**) horizontal polymerization (self-assembly resulting in a 2D layered structure) and (**b**) vertical polymerization (3D structure); (**c**) shows the result of molecular docking studies, revealing the most preferred orientation of an AzPTMS molecule placed on top of a layer formed by 9 other silane molecules.

The precise determination of the structure of the silane multilayer is not straightforward. Depending on the reaction conditions, the chemistry of the organosilanes and the type of the surface, a variety of structures can be formed, such as 2D layers (self-assembly) (Figure 7a), covalently-bonded layers and 3D surface polycondensates (Figure 7b). The uniform height of the silane film observed with AFM suggests that the growth proceeds through the (a) path, however, the thickness of the layer and the presence of additional agglomerates points to the vertical polymerization mechanism (b). Even though silane molecules are believed to cross-link through the formation of inter-molecular chemical bonds, the structure of the silane multilayer may be additionally determined by physical interactions, such as electrostatic or van der Waals forces. In order to determine to preferred orientation of the second silane molecular layer on top of the first one (the one bonded to glass), we performed molecular docking studies in which we looked for an optimal spatial arrangement of a silane molecule located close to the surface of a silane layer formed by 9 molecules exposing azide groups. Multiple models were obtained, with the most probable one including the location of azide groups of an incoming silane molecule in between the azide groups of molecules forming the initial layer (Figure 7c). The additional molecule in the second layer is, thus, oriented “upside-down” compared to those in the first layer. In such a configuration, the electrostatic potential created by the chemical groups located on the exposed (Si-) side of the second layer would form a network of positive-negative-positive charges (positive charges on hydrogen atoms in CH_3_ groups and negative charges on oxygen atoms). This could force the molecules in the eventual third layer to bind to the second one in a similar way the first one binds to glass (with the Si- end), adopting its positive-negative-positive “checkboard” to that formed by the second layer (positive-to-negative). What is more, such a configuration of the first two layers could lead to charge redistribution within the azide groups, resulting in shifting of the XPS N 1s peaks to lower binding energy values (as observed in our experiments).

**Figure 8:**
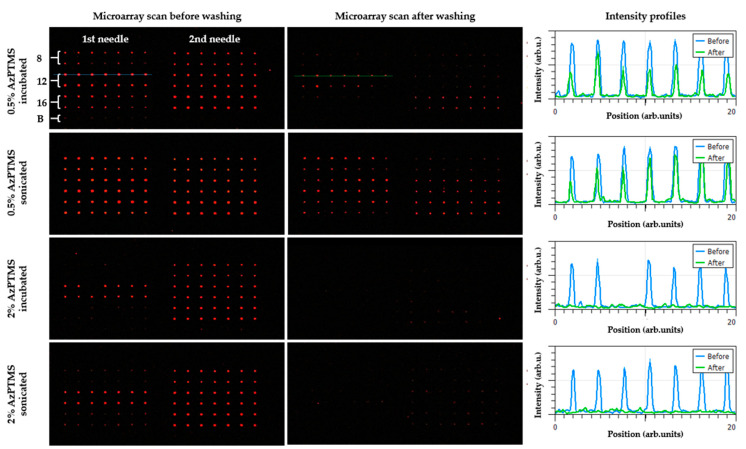
Comparison of microarray scans recorded for ODNs immobilized on glass slides functionalized with AzPTMS using incubation or sonication and 0.5% or 2% silane concentration. The (**left**) panel presents the microarrays just after printing, while the (**middle**) one following additional washing with 1% SDS. The printing was performed according to the scheme presented in Figure 3. The (**right**) panel shows intensity profiles taken over the 1st needle/third row spots in each image (exemplary rows are marked in the top row microarray scans with dotted blue and green lines).

Finally, we present the result of the printing of ODN probes (according to the scheme shown in Figure 3) on slides prepared in different ways (incubation or sonication, 0.5% or 2% solution of AzPTMS). The ODNs solutions A1–A3 were prepared shortly before immobilization. Figure 8 presents the images obtained just after printing (left panel) and following additional washing with 1% SDS (middle panel). The intensity scans (right panel) were taken over the 1st needle/third row probes in each image.

As it can be noticed, just after printing and before washing, the fluorescence of oligonucleotide probes is clearly visible for all the slides (in particular, for the 2nd needle print). Moreover, the spots are characterized by regular round shapes and good intensities. The process of microarrays washing decreased the spots’ fluorescence level on the sonicated 0.5% slides, however, not as significantly (Figure 8, right panel). For incubated 0.5% slides, the decrease in the spots intensity is much more pronounced. The probes on slides silanized in 2% solution of AzPTMS, both incubated and sonicated, are completely washed away. Thus, the best result was obtained for slides sonicated in 0.5% solution of AzPTMS.

The reason for the satisfactory binding efficiency of the silane layer prepared by sonication in 0.5% solution of AzPTMS can be rationalized based on the results of structural studies. AFM revealed that the as-prepared slide is the roughest one, so it may be concluded that the rougher the surface, the better the binding of nucleic acids. This may be related to the fact that clusters which stick out from the surface, contributing to the increased surface roughness, constitute silane agglomerates. According to the scheme shown in Figure 7b, the growth of agglomerates through the vertical polymerization mechanism may lead to a more pronounced exposure of azide groups, giving nucleic acids better binding access. This is in agreement with the XPS results which indicated a detectable amount of azide groups for this type of surface. In fact, an imprinted ODNs probe is a macroscopic object the bonding of which may be determined by macroscopic surface properties (such as, for example, roughness). Figure 4h presents an AFM image of an exemplary imprinted ODN probe—even though the frame size was set to the maximum allowed by this particular microscope (93 × 93 µm^2^), only a small fraction of probe is visible, revealing its dimensions (approximately 100 µm in diameter). The fact that the azide-related XPS signal was generally low (detectable only for the 0.5% slide) and that the 2% slides were not binding the ODN probes efficiently, may be related to the multilayer character of the silane layer, revealed by AFM and XRR, as well as the mutual orientation of adjacent layers (azide-to-azide configuration determined through the molecular docking studies). Such “upside-down” orientation of silane molecules at the surface layer may make the azide groups inaccessible to the ODN probes. In addition, it may lead to a charge redistribution within the azide groups through electrostatic interactions and promote shifting of the XPS N 1s peak positions to lower binding energy values. In such a scenario, only the clusters grown on top of the well-organized layer through the vertical polymerization mechanism, constituting silane agglomerates, would exhibit click-active azide groups.

## 4. Conclusions

We studied the surface structure of glass slides silanized by incubation or sonication in 0.5% or 2% solution of 3-azidopropyltrimethoxysilane, as well as their efficiency in binding, through a “click” reaction, oligonucleotide probes functionalized with a pentynyl linker. Atomic force microscopy revealed that independently of the preparation conditions silanization results in the formation of a continuous silane layer with occasional additional clusters and holes that are 2–3 nm deep. The layer prepared by sonication in a 0.5% silane concentration exhibited the highest surface roughness. X-ray reflectometry indicated that the total thickness of the layer is roughly double the height of the holes observed with atomic force microscopy, i.e. approx. 5 nm. X-ray photoelectron spectroscopy confirmed the expected increase of the amount of nitrogen at the surface after silanization, indicative for the formation of a silane layer. However, only the spectrum obtained for the slide silanized in a 0.5% silane concentration was hosting a detectable amount of azide groups. Low N 1s signal may be related to a partial neutralization of charge on nitrogen atoms via electrostatic interactions between azide groups through their mutual arrangement in densely-packed silane layers, as determined based on molecular docking studies. Best oligonucleotide binding efficiency was observed for the slides sonicated in 0.5% solution of 3-azidopropyltrimethoxysilane. This is believed to be related to the high roughness of the layer resulting from the vertical polymerization mechanism which leads to the formation of silane clusters with exposed azide groups.

## Figures and Tables

**Table 1:** The sequences of synthesized ODNs together with calculated and measured (MS) atomic mass values.

ODN	Sequence 5′–3′	Calculated Mass (amu)	Measured Mass (amu)
ODN-8	F AAC GGA GA Q	3230.8010	3246.694 [M+NH3-H]-
ODN-12	F AAC GGA GAT GGT Q	4496.9981	4576.602 [M+2K-3H]-
ODN-16	F AAC GGA GAT GGT TAT TQ	5722.1938	5788.848 [M+3Na-4H]-

**Table 2:** Contact angle values obtained for a clean glass slide and slides functionalized with 0.5% and 2% solutions of AzPTMS through incubation or sonication.

Concentration of AzPTMS (*v*/*v*%)	Silanization Process	Contact Angle (°)
Clean glass slide	–	<10
0.5	Incubation	69.5 ± 4.36
0.5	Sonication	67.1 ± 1.04
2	Incubation	78.8 ± 2.39
2	Sonication	78.6 ± 1.57

## Data Availability

Not applicable.

## Supplementary Materials

The following are available online at https://www.mdpi.com/article/10.3390/ma14112855/s1. Figure S1. FT-IR spectrum of 3-azidopropyltrimethoxysilane, Figure S2. ^1^H NMR spectrum of 3-azidopropyltrimethoxysilane, Figure S3. ^13^C NMR spectrum of 3-azidopropyltrimethoxysilane, Figure S4. ^29^Si NMR spectrum of 3-azidopropyltrimethoxysilane, Figure S5. HPLC chromatogram of ODN-8, Figure S6. HPLC chromatogram of ODN-12, Figure S7. HPLC chromatogram of ODN-16, Figure S8. MS TOF spectrum of ODN-8, Figure S9. MS TOF spectrum of ODN-12, Figure S10. MS TOF spectrum of ODN-16.
