# Supplementary material for: Structure and Oligonucleotide Binding Efficiency of Differently Prepared Click Chemistry-Type DNA Microarray Slides Based on 3-Azidopropyltrimethoxysilane
Source: Materials (Basel). 2021 May 26;14(11):2855. doi: 10.3390/ma14112855 (PMC8199275; doi:10.3390/ma14112855)
Supplement: Supplementary file 1 [file materials-14-02855-s001.zip › materials-1187620-supplementary.pdf]

Supporting Information

Structure and Oligonucleotide Binding Efficiency of Differently Prepared Click Chemistry-Type DNA Microarray Slides Based on 3-Azidopropyltrimethoxysilane

Emilia Frydrych-Tomczak [1], Tomasz Ratajczak [2], Łukasz Kościński [3], Agnieszka Ranecka [3], Natalia Michalak [3], Tadeusz Luciński [3], Hieronim Maciejewski [1,4], Stefan Jurga [5], Mikołaj Lewandowski [3,5,*] and Marcin K. Chmielewski [2,*]

[1] Poznań Science and Technology Park, Adam Mickiewicz University Foundation, Rubież 46, 61-612 Poznań, Poland

[2] Institute of Bioorganic Chemistry, Polish Academy of Sciences, Noskowskiego 12/14, 61-704 Poznań, Poland

[3] Institute of Molecular Physics, Polish Academy of Sciences, M. Smoluchowskiego 17, 60-179 Poznań, Poland

[4] Faculty of Chemistry, Adam Mickiewicz University, Uniwersytetu Poznańskiego 8, 61-614 Poznań, Poland

[5] NanoBioMedical Centre, Adam Mickiewicz University, Wszechnicy Piastowskiej 3, 61-614 Poznań, Poland

[*] Correspondence: chmielewskimk@ibch.poznan.pl, lewandowski@amu.edu.pl

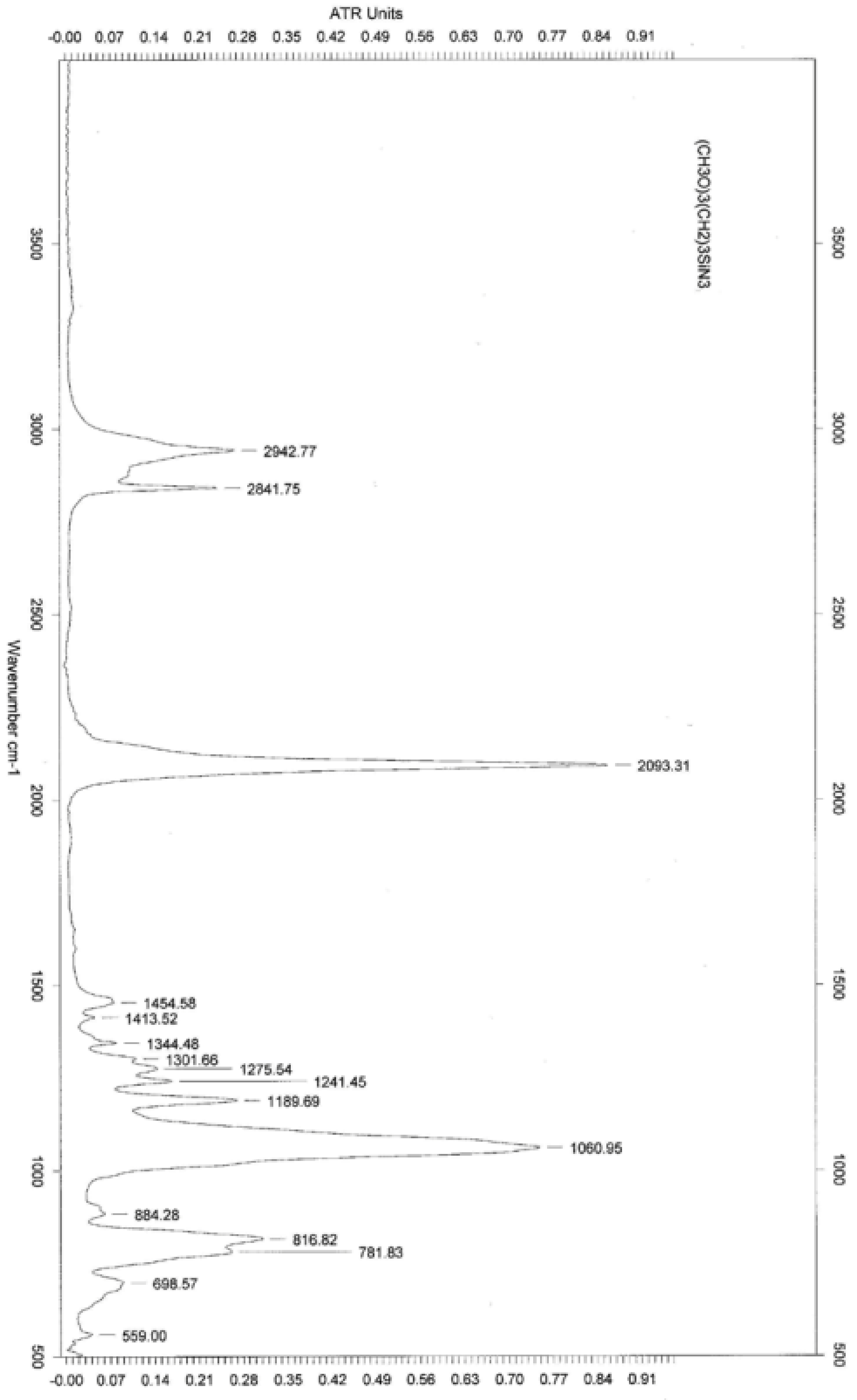

Figure S1. FT-IR spectrum of 3-azidopropyltrimethoxysilane.

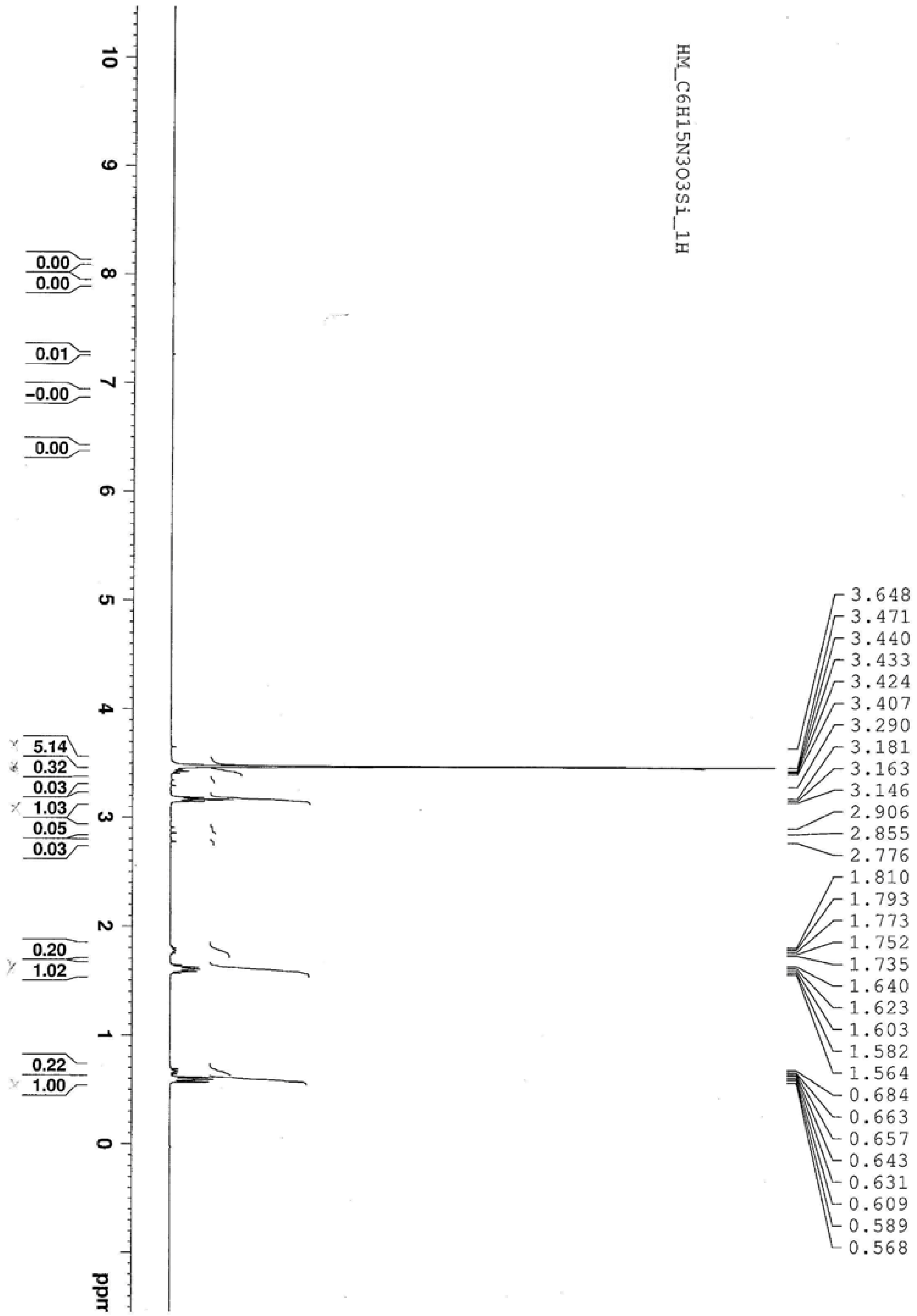

Figure S2. ^{1}H NMR spectrum of 3-azidopropyltrimethoxysilane.

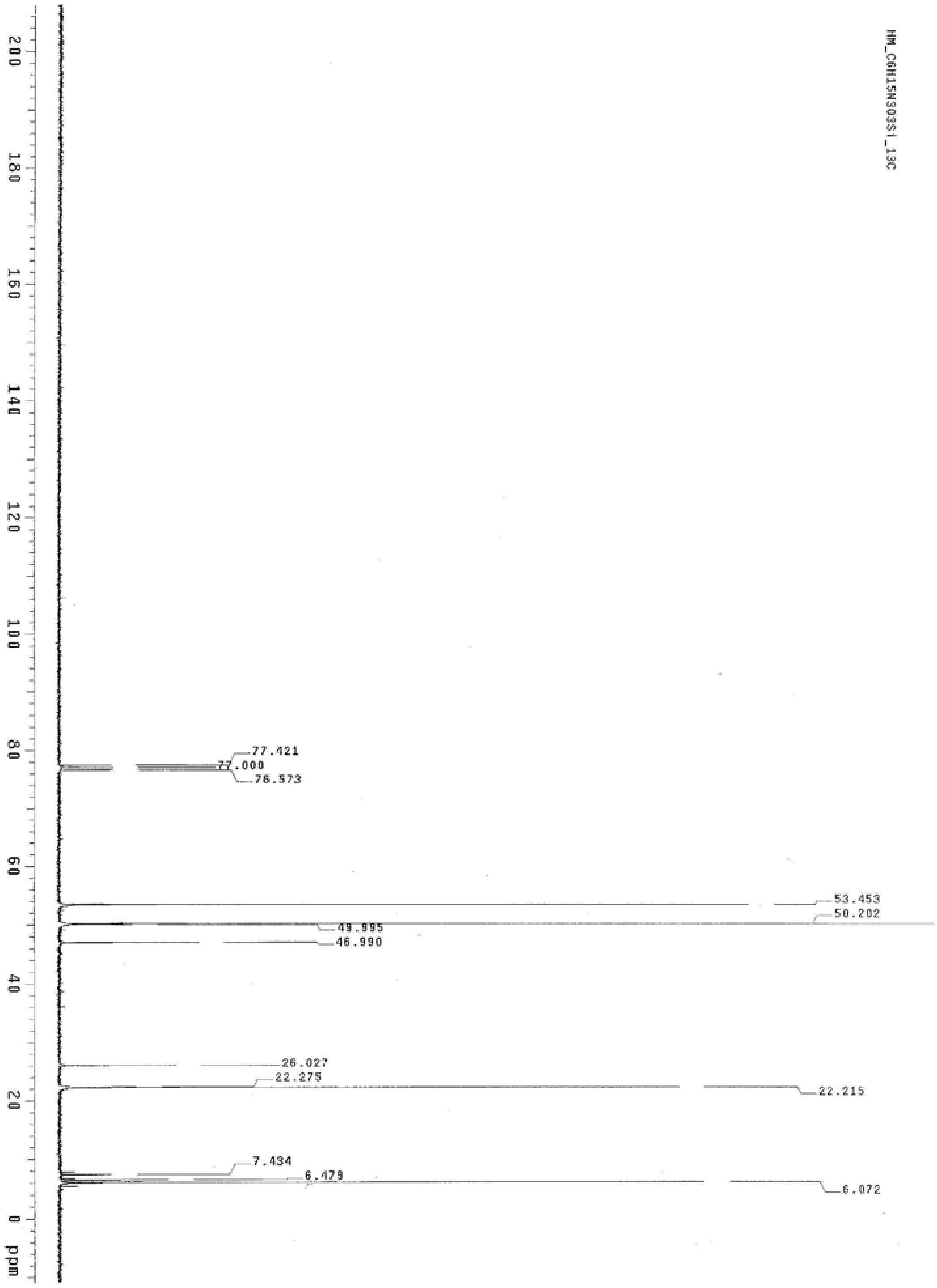

Figure S3. ^{13}C NMR spectrum of 3-azidopropyltrimethoxysilane.

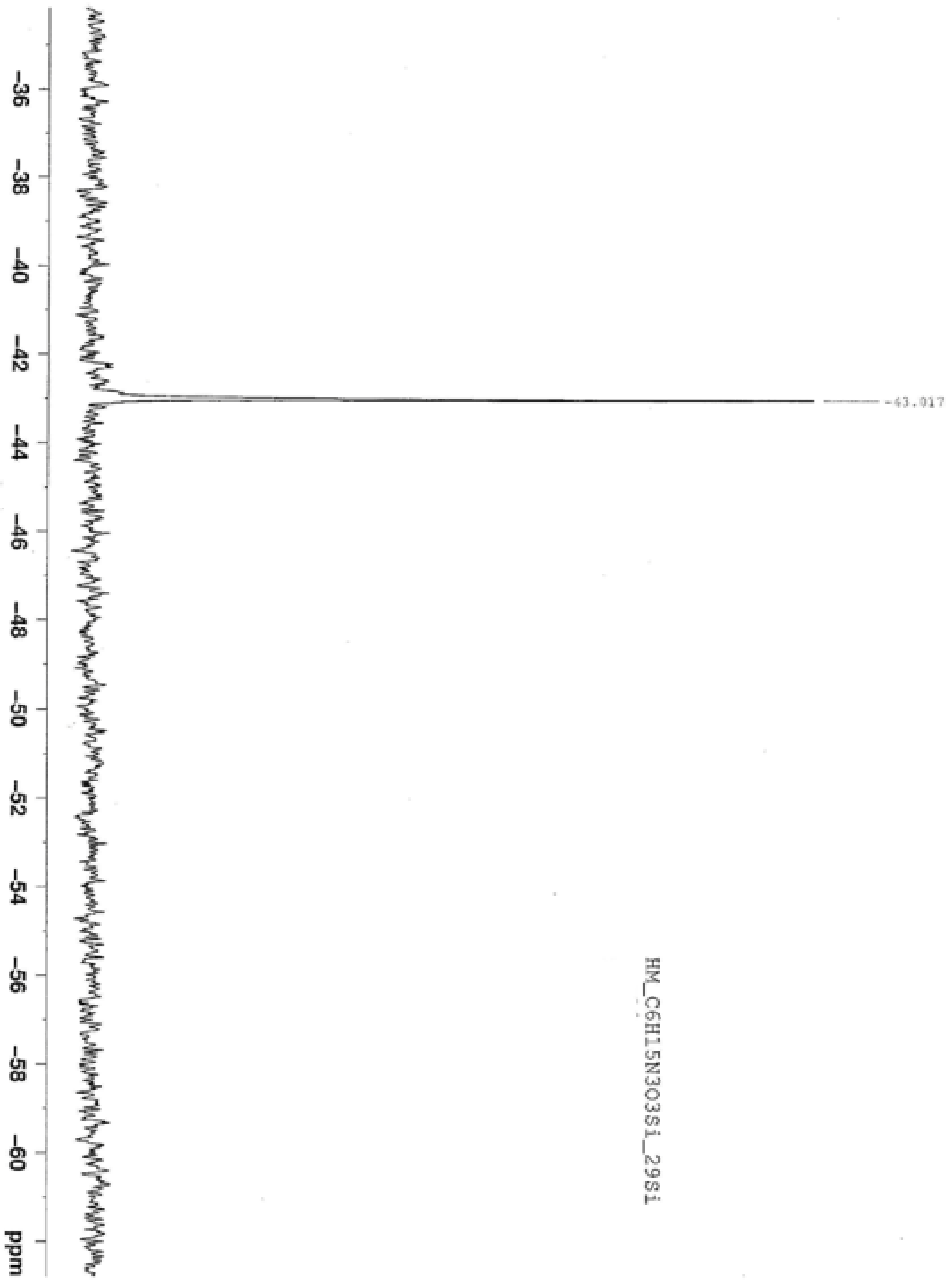

Figure S4. ^{29}Si NMR spectrum of 3-azidopropyltrimethoxysilane.

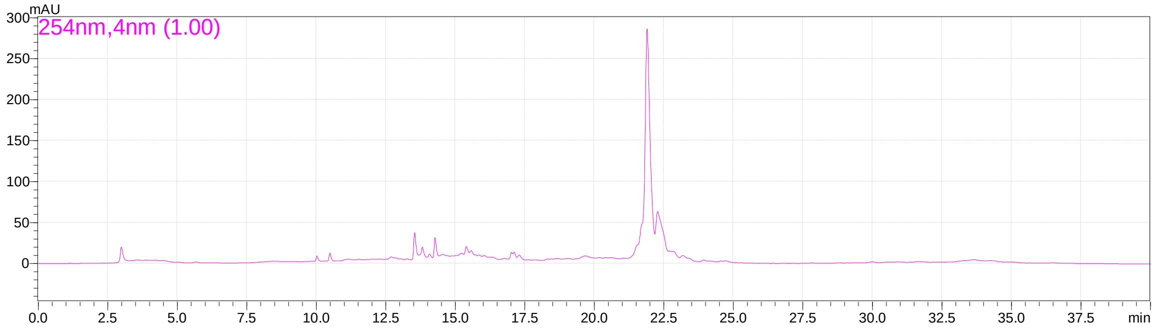

Figure S5. HPLC chromatogram of ODN-8.

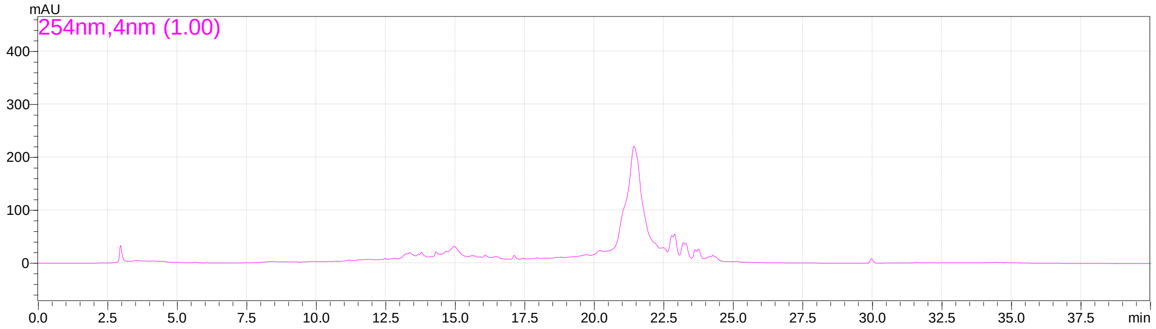

Figure S6. HPLC chromatogram of ODN-12.

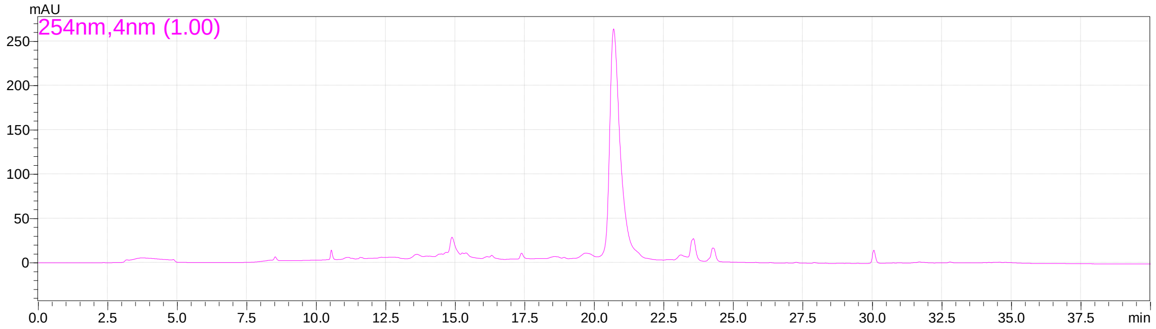

Figure S7. HPLC chromatogram of ODN-16.

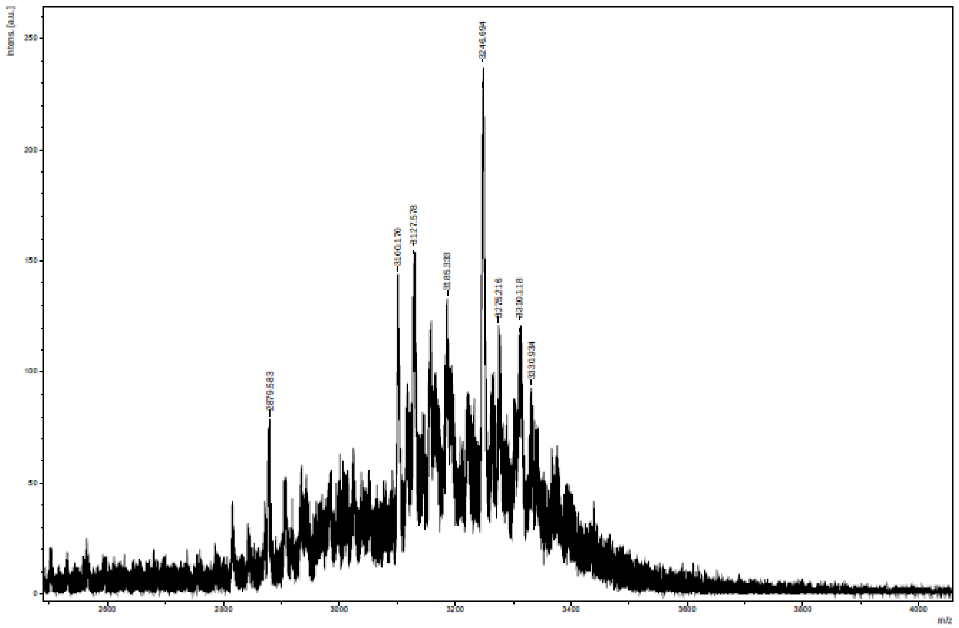

Figure S8. MS TOF spectrum of ODN-8.

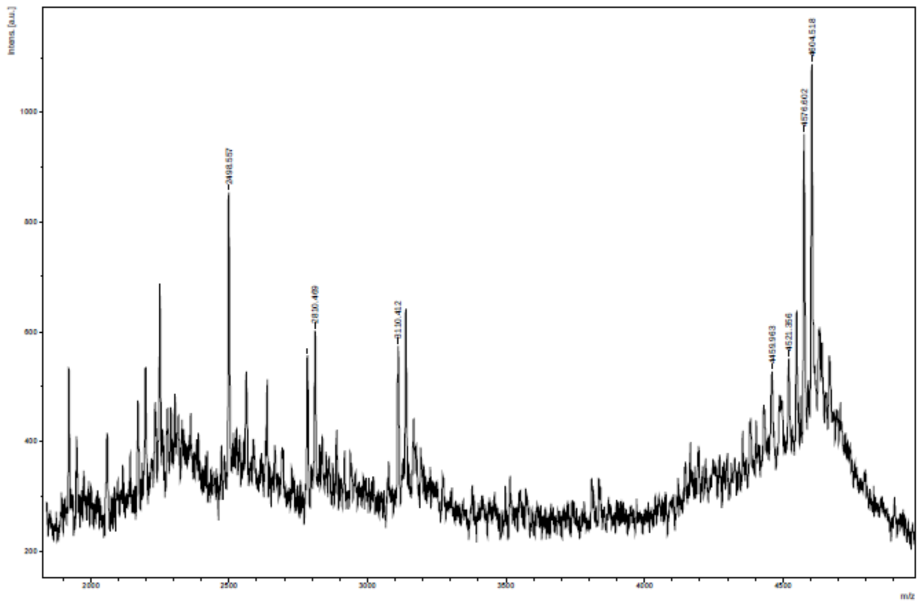

Figure S9. MS TOF spectrum of ODN-12.

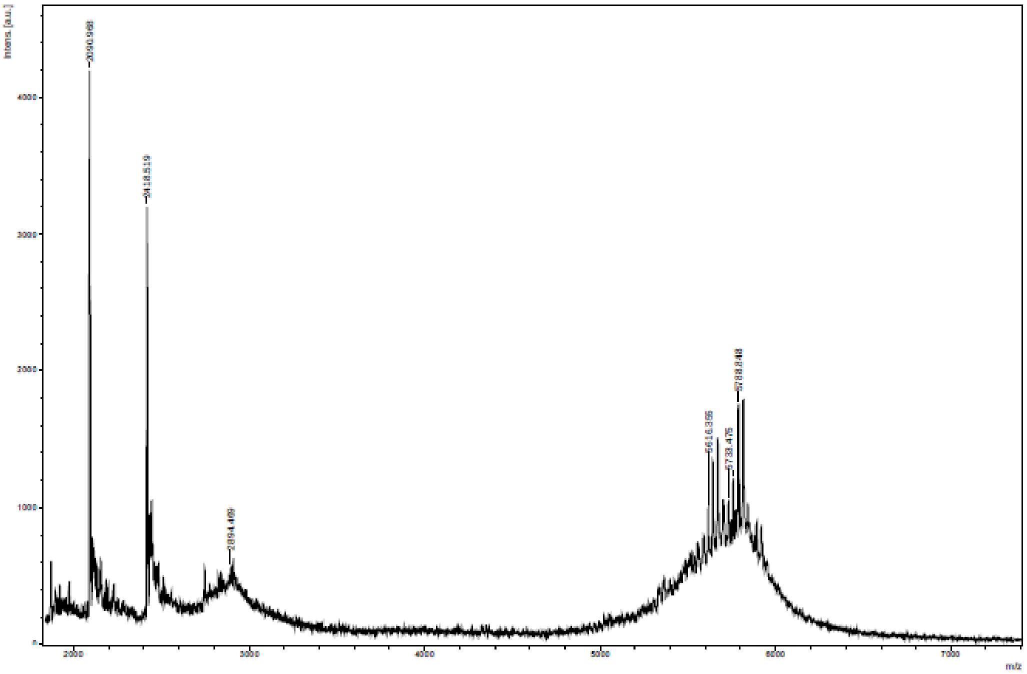

Figure S10. MS TOF spectrum of ODN-16.
